# Effect of Laser Power on the Corrosion and Wear Resistance of Laser Cladding TC4 Alloy

**DOI:** 10.3390/ma18245609

**Published:** 2025-12-14

**Authors:** Xiaolei Li, Sen Zhao, Kelun Zhang, Lujun Cui, Shirui Guo, Bo Zheng, Yinghao Cui, Yongqian Chen, Yue Zhao, Chunjie Xu

**Affiliations:** 1 School of Mechanical & Electronic, Zhongyuan University of Technology, Zhengzhou 450007, China; zsen1225@163.com (S.Z.); kelun522@163.com (K.Z.); cuilujun@126.com (L.C.); j10312@163.com (S.G.); zhengbo@zut.edu.cn (B.Z.); yh_cui@zut.edu.cn (Y.C.); chenyq@zut.edu.cn (Y.C.); 6941@zut.edu.cn (Y.Z.); 2 Zhengzhou Key Laboratory of Laser Additive Manufacturing Technology, Zhengzhou 450007, China; 3 School of Materials Science and Engineering, Xi’an University of Technology, Xi’an 710048, China; xuchunjie@xaut.edu.cn

**Keywords:** TC4 alloy, laser cladding, microstructure, corrosion resistance, frictional wear

## Abstract

TC4 alloy coatings were fabricated on a titanium alloy substrate using laser cladding. The influence of laser power ranging from 1000 W to 2200 W on the microhardness, wear resistance, and electrochemical corrosion behavior in 3.5% NaCl solution was systematically investigated. Results demonstrate that the TC4 coating exhibited a 35.17% enhancement in microhardness compared to the substrate, with an average value reaching 500 HV. As the laser power increased from 1000 W to 2200 W, the maximum wear depth progressively decreased, indicating significantly improved wear resistance, with fatigue wear being identified as the dominant mechanism. The coating prepared at 1400 W showed the best corrosion performance, displaying the highest self-corrosion potential of −0.110 V, the lowest corrosion current density of 0.125 μA·cm^−2^, and the largest polarization resistance of 2.057 × 10^6^ Ω·cm^2^. The charge transfer resistance initially increased and then decreased with increasing laser power. Numerical simulations revealed that when exposed to seawater, galvanic couples formed between the α and β phases on the TC4 titanium alloy surface, resulting in preferential dissolution of the β-phase.

## 1. Introduction

The study of laser cladding technology for titanium alloys began quite early in the 1980s and, after over half a century of development, it is now widely used in various fields and has become one of the major research focuses worldwide in the field of material surface modification [1]. Cladding is a quick process of melting and cooling; therefore, the entire process is relatively short and produces a closely bonded interface structure. Under certain conditions, an amorphous structure can be obtained, while the grains are effectively refined [2]. Compared to traditional surface modification techniques, laser cladding technology can result in less deformation of the processed surface, a smaller heat-affected zone, and control over the composition and dilution of the clad layer. In marine environments, the erosion and wear of metal and alloy structural materials is one of the critical factors limiting the reliability and lifespan of marine engineering equipment [3,4]. In fact, studies have confirmed that in terms of resistance to seawater corrosion, titanium and its alloys have better performance than stainless steel and copper alloys, hence they are referred to as “marine metals” [5]. However, in marine engineering applications, titanium and its alloys not only have to withstand the corrosion of seawater but also face mechanical effects such as friction and erosion. This situation may often lead to unexpected emergency failures, causing significant losses [6,7]. Therefore, it is particularly important to further study the friction loss behavior and mechanism.

Dong Yuecheng et al. [8] focused on the failure of marine equipment in harsh marine environments and conducted a study on the failure modes of TC4 ELI titanium alloy. They discovered that titanium alloys with different organizational structures possess different excellent material properties. Notably, titanium alloys with a net basket structure have better compressive creep properties, bi-modal structured titanium alloys have excellent low-cycle fatigue performance, and titanium alloys with a Weise formation have stronger stress corrosion resistance. Nabhani M et al. [9] used Ti-6Al-4V powder on a Ti-6Al-4V matrix to produce a fine needlelike martensite. This structure, being rather dense, possesses good chemical activity, thus significantly enhancing the corrosion resistance of the coating. Their research found that the TC4 coating produced by laser cladding simultaneously exhibited equiaxed crystals and fine needle-like structures, and good metallurgical bonding was formed at the bottom of the cladding layer with the base material. P. Henry et al. [10] studied the phenomena of friction corrosion in 316 stainless steel and TA6V4 titanium alloy in a sulfuric acid solution. They conducted a wear experiment on the 316 L stainless steel and TA6V4 titanium alloy metal panels and the abrasive material (polycrystalline alumina) under the conditions where sulfuric acid was the medium, through a friction wear tester, and connected to an electrochemical workstation during the experiment. They discovered that the coefficient of friction gradually decreases with increasing applied load, and the potential greatly affects the friction coefficient and wear resistance of the material. For TA6V4 alloy, the brittle and low-resistance films formed at the cathode or anode may lead to a high wear rate in the friction process. The formation and removal of these films result in the gradual consumption of the material.

However, there is a lack of systematic research on the corrosion and wear resistance of laser cladding TC4 alloy in an underwater environment [11,12,13,14]. In this study, the electrochemical corrosion behavior and friction and wear mechanisms of TC4 alloy formed under different laser powers in an underwater environment are studied, and the corrosion and wear mechanisms of the alloy are analyzed. It is expected to provide a theoretical reference for the application of laser cladding TC4 alloy in an underwater environment.

## 2. Experimental Procedures

### 2.1. Experimental Materials and Equipment

**Figure 1:**
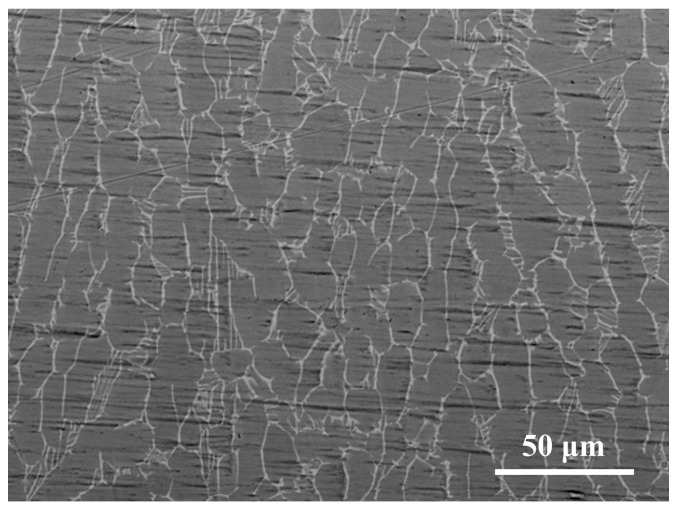
SEM micrograph of TC4 substrate surface.

The experiment selected a TC4 alloy (Ti-6Al-4V) with dimensions of 200 mm × 50 mm × 20 mm as the base material (Shanghai Yingxiong Metal Material Co., Ltd., Shanghai, China). Its physical properties and chemical composition can be found, respectively, in Table 1 and Table 2. As shown in Figure 1, about 30% of equiaxed α phase is distributed on the β phase transformation matrix. This microscopic structure endows the base material with good plasticity, elongation, and a high cross-sectional shrinkage rate. In order to make the cladding layer exhibit superior performance, first, the oxide film on the surface of the base material is removed using 400 mesh sandpaper. Ultrasonic cleaning was performed using deionized water to prevent penetration of foreign components, ions, salts, and oxides into the treated surface.

**Figure 2:**
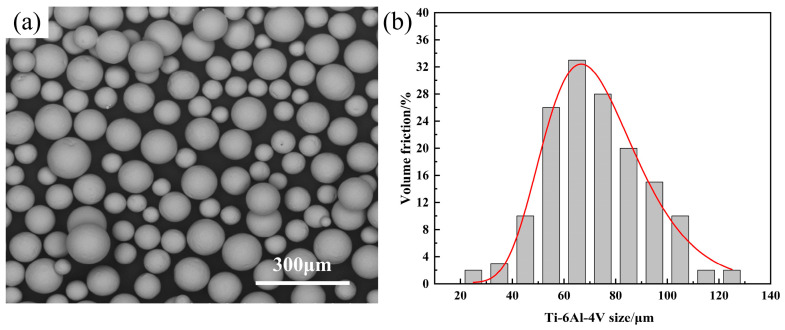
(**a**) TC4 powder; (**b**) particle size distribution of powder.

The TC4 powder used in this study was supplied by Xi’an Ouzhong Materials Technology Co., Ltd (Xi’an; China). Its main chemical composition is listed in Table 3. The powder has high purity and good sphericity, with an average particle size of 63.5 µm (Figure 2). To ensure the required powder fluidity, powder was dried in a vacuum oven (DZF-6050TE, Wuxi Marite Technology Co., Ltd., Wuxi, China) at 120 °C for 4 h to reduce moisture content by 99%.

**Figure 3:**
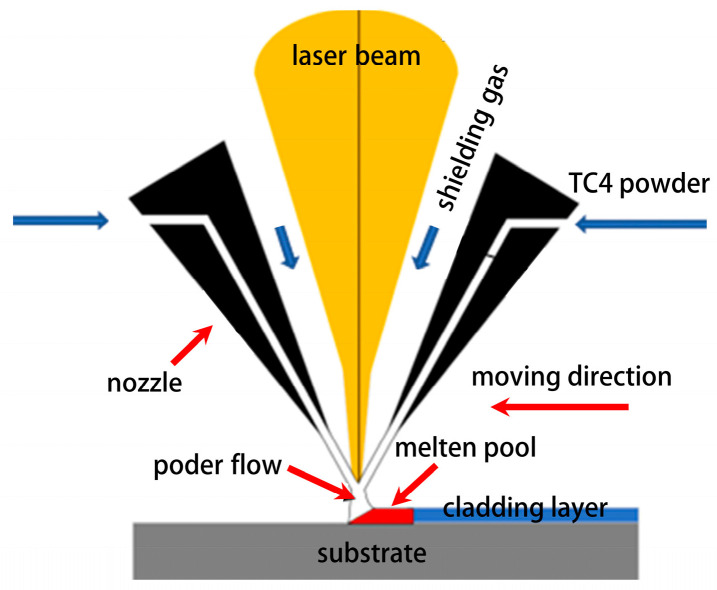
Schematic diagram of the laser cladding process.

The laser cladding experiment used the LDM8060 coaxial powder feeding system produced by Nanjing Zhongke Raycham Laser Technology Co., Ltd. (Nanjing, China). The system is equipped with an LDF-400-30 VG640 high-power semiconductor fiber-coupled laser (Lizerlane Laser Technology Co., Ltd., Shanghai, China). The laser was operated in continuous wave (CW) mode throughout this study. Its output laser wavelength is 1064–1070 nm, and the spot diameter on the substrate is 3 mm. To prevent alloy oxidation during the cladding process, the experiment is conducted under an argon-protected atmosphere. Figure 3 shows the principle of laser cladding processing. Based on the results of preliminary experiments, this study set the linear travel speed of the laser beam relative to the substrate surface at 480 mm/min and the powder feeding rate at 5.32 g/min, in order to achieve a stable melt pool and a consistent clad aspect ratio. The laser power is set to 1000 W, 1400 W, 1800 W, and 2200 W for cladding experiments. Each single-track cladding experiment was carried out over a length of 60 mm, resulting in a processing time of 7.5 s per track. The processing duration was primarily limited by the dimensions of the substrate and the need to avoid heat accumulation effects that could alter the cooling conditions. A 30 s interval was applied between successive tracks to allow the substrate to cool near room temperature, thereby ensuring consistent thermal conditions across all experiments.

### 2.2. Performance Testing

The hardness of a material is an important indicator of its ability to resist surface deformation or scratching. Generally, the higher the hardness, the better the wear resistance of the material [15]. The micro-hardness of the polished samples was tested on the cross-section under the HVS-5Z (Shanghai Fuley Measuring Equipment Co., Ltd., Shanghai, China) digital Vickers hardness machine to evaluate the hardness characteristics of the cladding layer. The load weight was 2.94 N, and the loading time was kept to 10 s. The fusion line where the clad layer and the base material meet was the zero coordinate, upwards as the positive direction, downwards as the negative direction. During the testing process, there was a 100-micron distance between each testing point, and each testing position was measured three times, and the average value was taken. The WTM-2E (Lanzhou Zhongke Kaihua Technology Development Co., Ltd., Lanzhou, China) controlled atmosphere micro-friction wear tester was used for the friction-wear experiments on the cladding layer. During the experiment, the grinding material was GCr15 (Shanghai Zhaotie Metal Material Co., Ltd., Shanghai, China), the load was chosen to be 200 g, the motor speed was 300 r/min, the rotating radius was 2 mm, and the running time was 1–2 h. The microstructure was observed by the Hitachi TM4000 Plus (Hitachi High-tech Co., Ltd., Tokyo, Japan) scanning electron microscope. The RTS5060F model electrochemical workstation produced by Zhengzhou Shireisi Instrument Technology Co., Ltd (Zhengzhou, China). was used when carrying out the corrosion resistance tests. A 3.5% concentration of NaCl solution was chosen as the electrolyte in the experiment. The prepared sample surface with an exposed area of 1 cm^2^ was used as the working electrode. Saturated mercurous sulfate was used as the reference electrode. A platinum sheet with dimensions of 10 mm × 10 mm × 0.2 mm was used as the counter electrode. For the potentiodynamic polarization tests, the potential sweep rate was 0.5 mV/s, with a sweep range from −1.0 V to +1.0 V.

## 3. Results and Discussion

### 3.1. Microhardness Analysis

**Figure 4:**
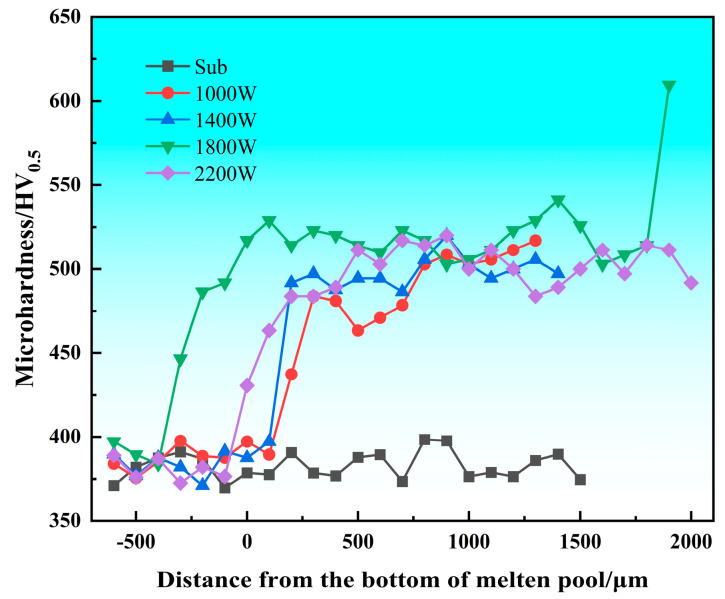
Hardness distribution curves of the cladding layer and substrate.

The microhardness of the cladding layer is an important indicator for evaluating the quality of cladding. From the results (in Figure 4), it can be clearly seen that the hardness of the coating after cladding is significantly higher than the hardness of the substrate. The hardness of the cladding layer after laser cladding is about 35.17% higher than that of the substrate, and the average hardness of the cladding layer can reach about 500 HV. According to the trend of the hardness curve of the cladding layer, we found that the curve is roughly a step-like distribution, and the micro-hardness near the heat-affected zone is slightly higher than the substrate part. This is due to the hard phase in the cladding layer diffusing to the heat-affected zone, and due to rapid cooling under laser remelting, a quenching effect occurs, thus increasing hardness [16]. By comparing the hardness curves of the cladding layer under different process parameters, it can be found that the hardness changes smoothly inside the cladding layer, while the hardness value of the material in the heat-affected area decreases in a stepwise manner. This phenomenon occurs because part of the heat is absorbed by the substrate during the high-energy laser scanning process, which causes the substrate to have a quenching and strengthening effect. This absorbed energy decreases as the depth of the substrate increases, resulting in a diminishing strengthening effect. The cladding layer is able to observe three zones, namely the cladding zone, the metallurgical bonding zone, and the matrix heat-affected zone. From the observation results, the hardness can reach 525 HV at the top of the cladding layer, and the hardness drops sharply to 450 HV at the junction line. The microhardness of the heat-affected zone of the matrix is between 400 and 450 HV, and this part of the matrix is affected by the laser heat, and the grains are refined to form martensite [17]. Therefore, the microhardness has been significantly improved compared with the matrix.

**Figure 5:**
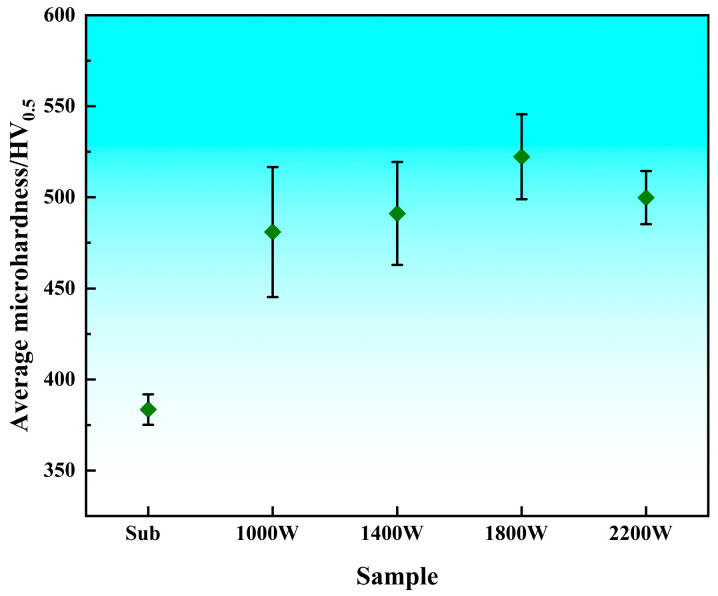
Average hardness of the cladding layer and substrate.

Figure 5 shows the average hardness of the cladding layer and substrate of a single-pass TC4 alloy under different process parameters. By analyzing the average and standard deviation of the hardness of several specimens, we can see that the microhardness of the coating surface is significantly improved by laser cladding technology. When the laser power is 1800 W, the average hardness is the highest, and the standard deviation of the hardness in the cladding layer is moderate, indicating that the hardness distribution in the cladding layer is relatively uniform. On the contrary, when the laser power is 1000 W, the average hardness is lower, and the internal hardness of the cladding layer fluctuates the most, but the cladding layer has no defects such as cracks and porosity. When the laser power is 2200 W, it is the smallest set of hardness standard deviations in several cladding experiments, and the hardness distribution inside the cladding layer is the most uniform.

### 3.2. Cladding Layer Friction and Wear Experiment

**Figure 6:**
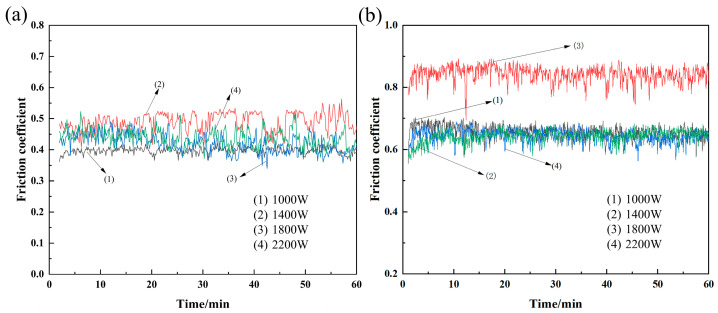
Coefficient of friction curve of the specimen: (**a**) underwater environment; (**b**) air environment.

In order to simulate the underwater environment as much as possible in the tribo-wear experiment with 3.5% NaCl solution as the medium, we can analyze the trajectory of the surface friction coefficient of each test sample as a function of time according to the friction coefficient chart shown in Figure 6. The initial trend of the wear curve is usually higher and then lower. In the initial wear test stage, the sample is in point-to-point contact with the grinding ball, and the two friction surfaces have not been thoroughly run in. In the running-in stage, the geometry of the two friction surfaces will change, and the shear force will be large, so that the friction coefficient will rise rapidly. After severe wear, the contact pattern gradually changes to face-to-face contact, at which point the coefficient of friction begins to gradually decrease and enters a relatively stable wear phase. In the stable wear phase, as the friction process progresses, wear particles are gradually generated, which accumulate in the friction area. When the abrasive particles accumulate to a certain extent, the frictional resistance between the friction surfaces increases, resulting in an increase in the friction coefficient. After the titanium alloy cladding layer enters the stable wear stage, the value gradually stabilizes at about 0.45, accompanied by fluctuations. However, in contrast, the value of the cladding layer in the air medium will increase suddenly in some areas after entering the stable wear stage (in Figure 6b) and finally stabilize in the range of 0.65–0.85, which is significantly higher than that of the cladding layer worn in the underwater environment.

**Figure 7:**
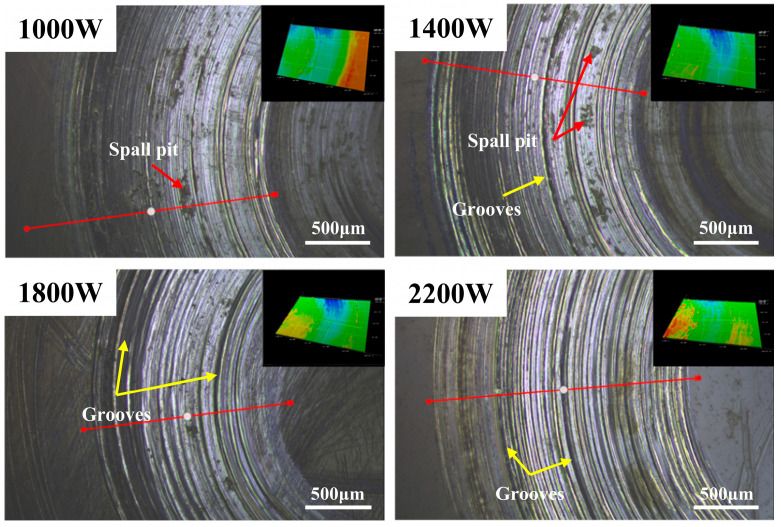
Three-dimensional topography of the wear area after sliding-wear tests for samples fabricated at different laser powers.

The sample after the friction and wear test in the seawater medium was removed, and after ultrasonic cleaning, it was placed under an ultra-depth-of-field optical microscope to observe the wear area. The ultra-depth-of-field microscope can show the topography of the surface of the specimen after wear. Figure 7 shows the the three-dimensional topography of the wear area.

**Figure 8:**
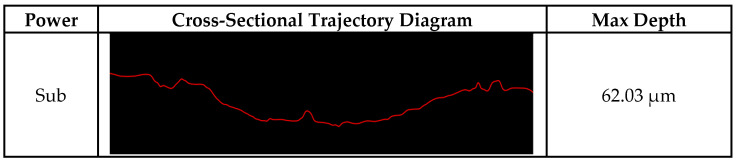

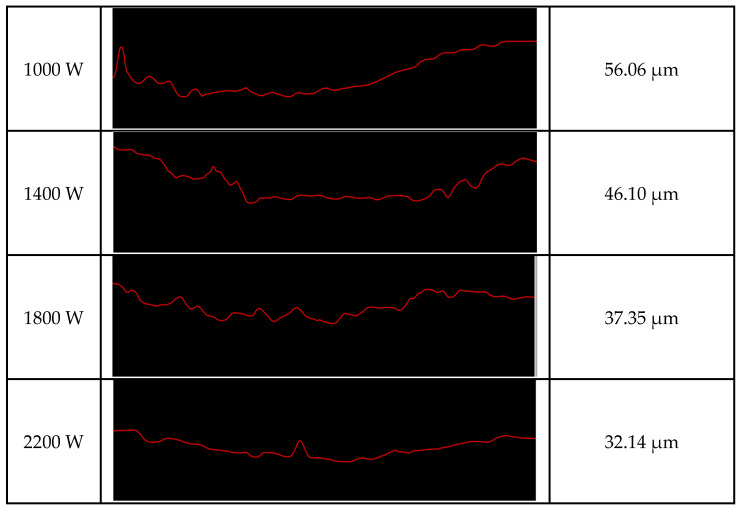
Wear profile curves of samples fabricated at different laser powers.

As can be seen in Figure 7, the height of the worn balls is significantly lower than that on both sides, and at the beginning of the experiment, the balls are in point contact with the surface of the material. As the experiment progressed, the surface of the specimen began to be dented by the ball and began to change from point contact to surface contact. Many grooves and abrasive debris appeared in the wear positions, some of which were formed by extrusion. Figure 8 shows the cross-sectional trajectory curve of the wear part.

**Figure 9:**
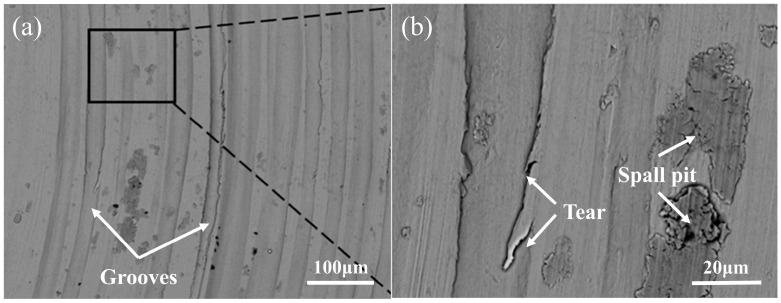
(**a**) Wear morphology of 2200 W power sample in seawater; (**b**) enlarged view of the boxed area in (**a**).

From the wear-profile curve images (Figure 8), it is evident that the wear resistance of the laser-clad specimens is markedly superior to that of the substrate. Moreover, compared with other processing parameters, the specimen fabricated with a laser power of 2200 W exhibits a shallower wear track. The inconsistency in the height can lead to large fluctuations in the coefficient of friction when performing friction and wear tests, even if the cladding layer has good wear resistance. In addition, the three-dimensional topography information is extracted to measure the groove depth resulting from material wear. The maximum wear depth of the cladding layer was measured to be 56.06 μm when the laser power was 1000 W. The wear depth of the cladding layer decreased by about 43% when the laser power was increased from 1000 W to 2200 W. It can be seen in the figure that on the surface of the cladding layer after wear, there are deeper grooves, more spalling pits, and more debris. Plastic deformation and delamination are severe, indicating that the wear form is fatigue wear. Figure 9 shows the SEM image of the wear area in seawater medium when the laser power is 2200 W. The surface of the cladding layer under this process contains a large amount of debris, with many shallow grooves formed by debris, increased plastic deformations, and wear that is mainly abrasive. It can be seen from the wear cross-section that the wear morphology is relatively gentle under this process, and there are no complex grooves and protrusions, which is related to the uniform hardness distribution inside the cladding layer. In the zoomed-in area of the SEM image, it can be seen that there are traces of tearing near the grooves, which is due to the high-speed rotation of the material and the extrusion and cutting effect of the grinding material. The coating formed under the 1800 W process has severe internal protrusions, which are partly related to its relatively high hardness and uneven hardness distribution.

### 3.3. Electrochemical Corrosion Behavior of Cladding Layers

#### 3.3.1. Potentiodynamic Polarization Curve

**Figure 10:**
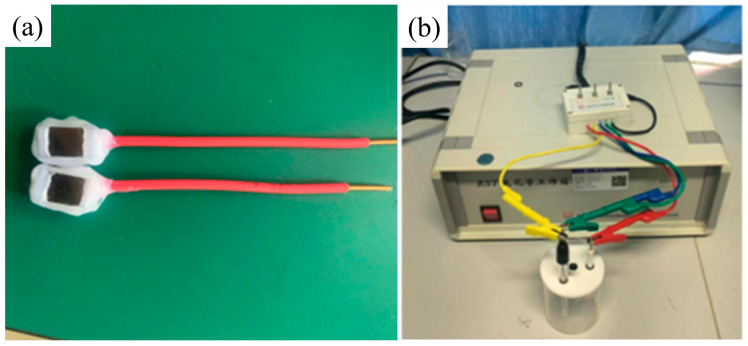
(**a**) Working electrode treatment; (**b**) connection diagram of electrochemical experiments.

**Figure 11:**
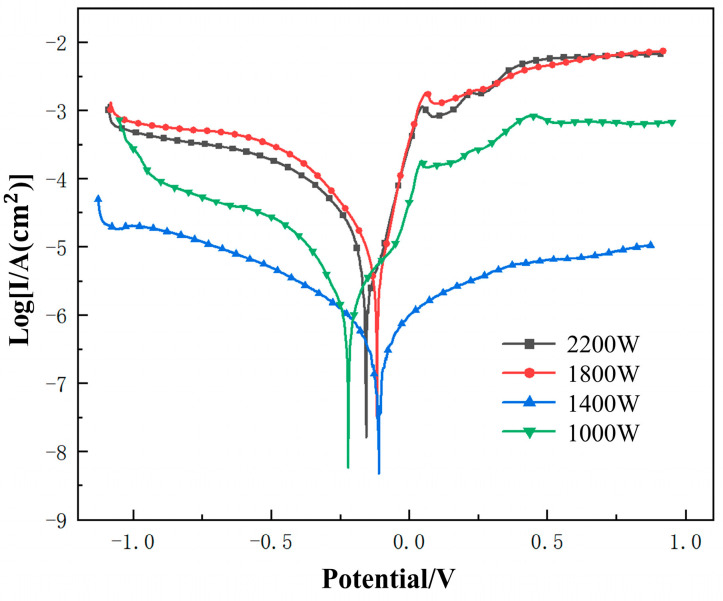
Potential polarization curves of the cladding layer and matrix in 3.5% NaCl solution.

In order to simulate the marine environmental conditions as realistically as possible, we used a three-electrode electrochemical system for our experiments. In this system, we choose a platinum sheet as the working electrode, saturated calomel as the reference electrode, and the workpiece to be tested is sealed as the working electrode, as shown in Figure 10a. Figure 10b shows the connected device. Using a 3.5% NaCl solution as the electrolyte, the potential polarization curves of the cladding layer and substrate in NaCl solution under different process parameters were studied at room temperature. The sweep range was selected as 0.2 V before and after the open circuit potential. As shown in Figure 11, the potential polarization curves of four samples are shown. Through the calculation of Tafel extrapolation, we can obtain the parameters of self-corrosion potential (E_corr_), corrosion current density (*i_corr_*), corrosion rate (*v_corr_*), and polarization resistance (*R_p_*).

When the electrode potential is very close to the open-circuit potential, the current-to-potential curve can be approximated as a straight line, which can be deduced by the Stern–Geary formula [18]:

(1) $$R_{p}=\frac{B}{i_{corr}}$$

(2) $$B=\frac{\beta_{a}\beta_{c}}{2.303\left(\beta_{a}+\beta_{c}\right)}$$

In the formula, *β_a_* is the anode slope of the Tafel curve, and *β_c_* is the cathode slope of the Tafel curve. It can be seen from the above equation that the polarization resistance is inversely proportional to the self-corrosion current density, and the larger the polarization resistance, the smaller the self-corrosion current density, and the better the corrosion resistance of the material [19].

In general, the corrosion rate (*v_corr_*) is more commonly used to determine the corrosion resistance of a material, and when uniform corrosion occurs on the surface of a material, it can be simply converted to the corrosion rate formula using the weight loss method as follows [20]:

(3) $$v_{corr}=\frac{K\times EW\times i_{corr}}{\rho }$$

In the above equation, *v_corr_* represents the corrosion rate (mm/a), *i_corr_* represents the corrosion current density (μA/cm^2^), *K* is the constant, generally 3.27 × 10^−3^, *EW* is the alloy equivalent, and *ρ* is the pattern density (g/cm^3^), which shows that there is a positive correlation between the corrosion rate and the corrosion current density [21]. Table 4 calculates the values of self-corrosion potential (E_corr_), corrosion current density (*i_corr_*), corrosion rate (*v_corr_*), and polarization resistance (*R_p_*) calculated by the Tafel extrapolation method and formula.

As can be seen from Figure 11, when the scanning voltage is lower than the self-corrosion voltage, the external power supply protects the anode part. In this case, electron enrichment occurs on the surface of the test workpiece and initiates a reaction of electrolysis of water. As the voltage slowly changes in the positive direction, the response of the working electrode system to the voltage increases, resulting in a slow decrease in the actual voltage of the electrochemical reaction at the interface, which is manifested as a decrease in the current at the macro level [22,23].

(4) $$2H^{+}+2e^{-}\to H_{2}$$

Once the voltage reaches the self-corrosion potential of the sample, almost all of the voltage is absorbed by the metal sample, resulting in a minimum current. At this point, the oxidation reaction of the working electrode begins. With the increase in the voltage on the working electrode, the electronic loss on the surface of the workpiece becomes more significant, resulting in an increase in the dissolution rate of the workpiece surface [24].

(5) $$Ti\to Ti^{2+}+2e^{-}$$

(6) $$Ti^{2+}+2H_{2}O\to Ti{\left(OH\right)}_{2}+2H^{+}$$

At this stage, as the current increases, the concentration of metal cations in the electrochemical system gradually accumulates to a certain extent. The working electrode is in a highly active state due to the lack of electrons, which causes further oxidation of the corrosion products already present in the electrochemical system to form a stable passivation film, known as surface passivation. By comparing the potentiodynamic polarization curves under different process conditions, it can be observed that the TC4 coating with laser cladding exhibits more significant passivation behavior than the substrate. In addition, the TC4 coating of laser cladding has a high self-corrosion potential, in which when the laser power is 1400 W, its self-corrosion potential reaches the highest value (−0.110 V), the corrosion current is (0.125 μA·cm^−2^), and the polarization resistance is the largest (2,056,570 Ω), which means that it performs well in corrosion resistance. However, under the process parameters of 1000 W, 1800 W, and 2200 W with laser power, the passivation area of the cladding layer is obvious, and the slope of the curve around the voltage of 0.7 V decreases sharply. Especially at the laser power of 1000 W, the curve even has a breakdown phenomenon in the second half, followed by secondary passivation [25].

#### 3.3.2. AC Impedance Spectroscopy

**Figure 12:**
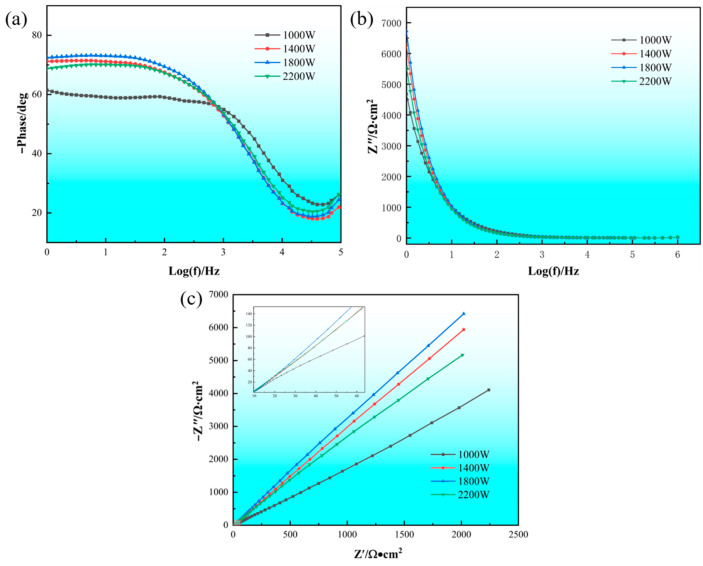
Electrochemical impedance: (**a**) and (**b**) Bode diagrams; (**c**) Nyquist diagram.

Under the conditions of scanning speed (480 mm/s) and powder feeding rate (5.32 g/min), the electrochemical impedance of samples with different powers (1000 W, 1400 W, 1800 W, and 2200 W) was tested, respectively. Figure 12 shows the Nyquist and Birdt diagrams of the four sample groups in 3.5% NaCl solution. When testing the electrochemical impedance of a material, the Nyquist diagram consists of the real part and the imaginary part of the impedance, and the larger the arc radius of the curve, the better the corrosion resistance of the specimen [26]. When the power rises from 1000 W to 1400 W, the corrosion resistance of the material is most obvious. Increasing the power from 1400 W to 1800 W improves the corrosion resistance of the material, and when the power is further increased, the corrosion resistance of the material decreases.

By analyzing the Bode diagrams (a) and (b), it can be seen that the Bode diagram is a function of frequency with respect to the phase angle and impedance modulus. For titanium coatings formed under different laser powers, the modulus value and phase angle of the impedance increase when the applied frequency f increases. When the laser power is 1800 W, the phase angle in the range of low to medium frequency is relatively large, which indicates that the laser cladding TC4 titanium alloy has a high capacitive behavior and forms a dense oxide film on the surface of the cladding layer [27]. Therefore, when the laser power is 1800 W, the laser cladding TC4 alloy cladding layer has the best corrosion resistance.

**Figure 13:**
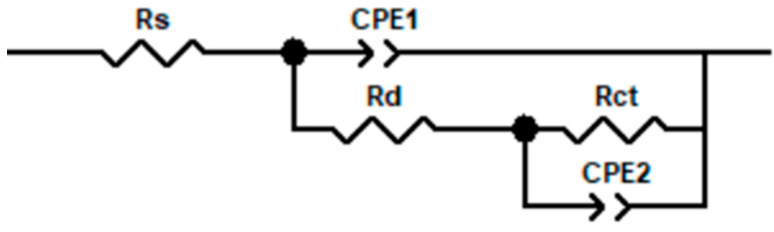
Equivalent circuit diagram.

The impedance spectra of the titanium alloy coating prepared under different laser powers in 3.5% NaCl solution are fitted, and the equivalent circuit diagram is shown in Figure 13. In the equivalent circuit model, Rs is the resistance caused by the electrolyte solution, Rd is the resistance caused by the surface defects of the cladding layer, Rct is the resistance caused by charge transfer, and CPE_1_ and CPE_2_ are two constant phase angle elements. If the dispersion index *n* = 1, the CPE behaves as an ideal capacitor, whereas when the value of n is between 0.5 and 1, the CPE is considered a non-ideal capacitor [28]. After fitting the impedance using ZView 3.1 software, the parameter values of each component in the circuit diagram can be obtained, as shown in Table 5.

The results showed that there was little change in the resistance of the electrolyte solution in the four groups of experiments. As the laser power increases, the Rct increases first and then decreases. When the laser power increases from 1000 W to 1800 W, the resistance value of Rct gradually increases, indicating that the passivation film produced on its surface gradually becomes complete and dense with the increase in power. When the laser power is 1800 W, the cladding layer has a maximum charge transfer resistance Rct ((5.8864 ± 0.22) × 10^5^), which is much higher than that of the other three groups of experiments. The dispersion index n can also reflect the density of the oxide film formed on the surface of the sample, and the laser power is 1800 W, which is the best density of the oxide film formed.

#### 3.3.3. Simulation Analysis of TC4 Titanium Alloy α and β Two-Phase Corrosion

**Figure 14:**
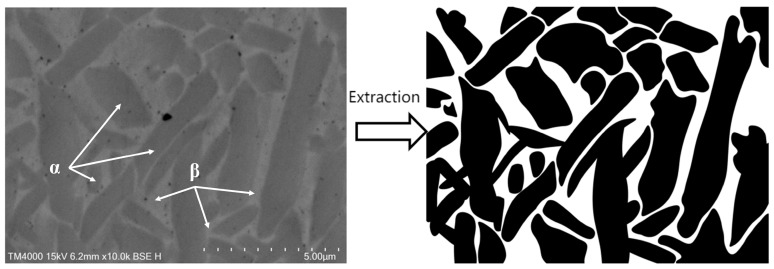
Tissue extraction steps.

**Figure 15:**
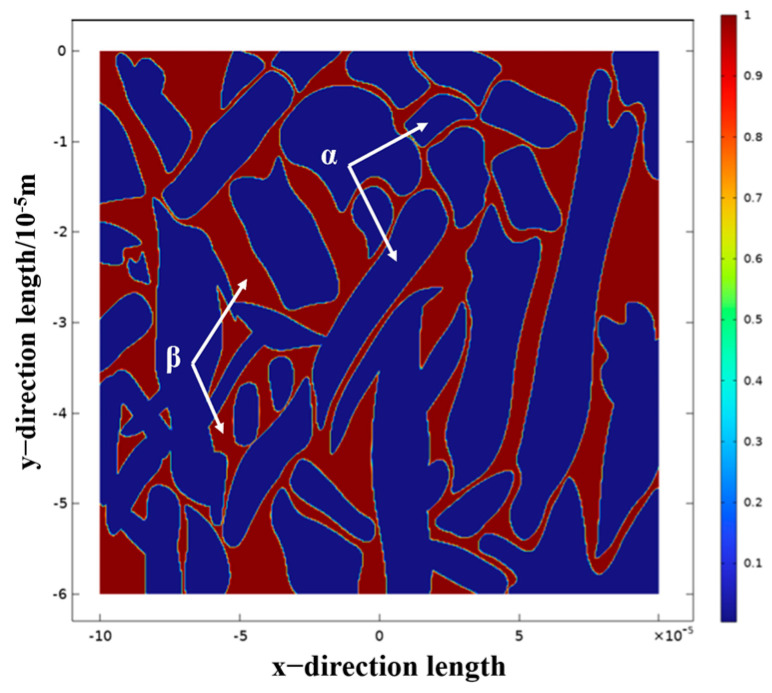
Microstructure model of TC4 alloy containing α and β cross-sections.

Laser cladding of titanium alloy will produce both α and β phases inside the cladding layer, and the corrosion potential of these two phases is different. When the sample is immersed in the electrolyte, a galvanic cell will be formed to accelerate the corrosion rate. In order to explore this corrosion mechanism, the COMSOL Multiphysics 6.1 software was used to perform electrochemical simulations, and the experimental α and β phases of TC4 titanium alloy and the microstructure distribution were used as boundary conditions to solve the Nernst–Planck equation, and the potential distribution and current density of the galvanic cell were solved. In order to identify the electrode boundaries of the α phase and the β phase, the tissue pictures taken by SEM were imported into Photoshop software to extract the tissue structure and converted into binary images to serve as the interpolation level set function L. Figure 14 below is the tissue extraction step, and Figure 15 is the microstructure model of the cross-section after import.

The current density at the electrode surface can be obtained by solving for the current and potential distributions in the electrolyte solution, and the migration of species *i* in the model is represented by the Nernst–Planck equation:

(7) $$\frac{\partial c_{i}}{\partial t}=-\nabla \cdot N_{i}=D_{i}\nabla^{2}c_{i}-z_{i}Fu_{i}\nabla \Phi$$

where *N_i_* is the flux, *z_i_* is the number of charges, *u_i_* is the mobility, Φ is the electrolyte potential, and F is the faraday constant.

In an electrolyte solution, the conservation of the mass of substance *i* can be expressed as:

(8) $$\frac{\partial c_{i}}{\partial t}=-\nabla \cdot N_{i}=D_{i}\nabla^{2}c_{i}-z_{i}Fu_{i}\nabla \cdot \left(c_{i}\nabla \Phi \right)+\nabla \cdot \left(c_{i}U\right)$$

Suppose that in this model: 1. the solvent is an incompressible liquid; 2. the solution is electrically neutral; 3. the electrolyte solution is well mixed and there is no concentration gradient; and 4. the hydrogen evolution reaction occurs on the cathode surface while the dissolution reaction occurs on the anode surface. Based on the above assumptions, the equation can be simplified to Equation (8).

The boundary conditions of the cathode surface and the anode surface can be expressed as:

(9) $$\nabla_{n}\Phi =-\frac{I_{c}\left(\Phi \right)}{\sigma }$$

(10) $$\nabla_{n}\Phi =-\frac{I_{c}\left(\Phi \right)}{\sigma }$$

In the above formula: σ represents the conductivity of the electrolyte solution, $I_{a}\left(\Phi \right)$ represents the current density of the anode, and $I_{c}\left(\Phi \right)$ and is the current density of the cathode.

The polarization curves of the two metals were measured by electrochemical experiments on near-α titanium alloy and near-β titanium alloy, and the piecewise linear interpolation functions $I_{a}\left(\Phi \right)$ and $I_{c}\left(\Phi \right)$ were obtained.

Electrode kinetics expressions are used to model the electrode reaction of the β phase on the electrode surface. The local current density of the β phase on the electrode surface is:

(11) $$I_{i\beta }=f\left(\Phi_{s,ext}-\Phi_{1}\right)\times \left(1-micro\left(x,y\right)\right)$$

**Figure 16:**
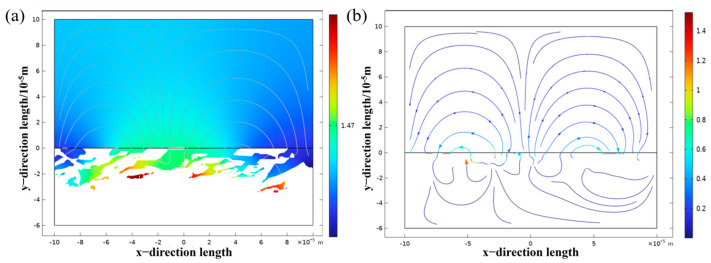
Simulation results: (**a**) numerical simulation results of potential; (**b**) current density simulation results.

Figure 16 shows the results of the numerical simulation of electrochemical corrosion, and it can be seen that when the galvanic cell is formed in the electrolyte by the α phase and the β phase, the potential of the β phase is lower than that of the α phase, so the electrolyte is always limited in the dissolution. Due to the high potential of the α phase, it belongs to the protected party, and no corrosion occurs.

The results of numerical simulation show that the current density of the β phase is larger than that of the α phase, and the β phase is more susceptible to corrosion. On the one hand, this is because the vanadium element in TC4 titanium alloy is mainly enriched in the β phase, and when in NaCl solution, the content of vanadium oxide in the passivation film formed on the β phase is relatively high, and vanadium oxide is relatively easy to dissolve in Cl ion solution. Therefore, when the α phase and the β phase form a galvanic cell in solution, the β phase will dissolve first. On the other hand, when corrosion occurs at grain boundaries, high dislocation density, low hardness, and elastic modulus, microcracks are first generated in the intergranular β phase, and then over time, the oxides expand and penetrate each other in the process, eventually leading to α grain peeling. When the α grains are peeled off, a new passivation film is formed on the new surface, and the new passivation film slows down the corrosion rate to a certain extent. The titanium alloy coating formed by laser cladding is mostly fine α needle-like martensite, which greatly improves the hardness and elastic modulus of the coating, and enhances the ability of the surface of the titanium alloy material to generate a passivation film in artificial seawater.

## 4. Conclusions

The microhardness and friction, and wear properties of TC4 coating prepared under different laser powers were analyzed, and the electrochemical corrosion behavior of TC4 coating in 3.5% NaCl solution was studied by electrochemical corrosion test. The α phase and β phase in the cladding layer were numerically simulated, and the following conclusions were drawn.

(1) The hardness of the cladding layer after laser cladding is about 35.17% higher than that of the substrate, and the average hardness inside the cladding layer can reach about 500 HV. The average hardness of the laser power of 1800 W is the highest, indicating that the hardness distribution in the cladding layer is relatively uniform. Conversely, when the laser power is 1000 W, the average hardness is lower, and the internal hardness of the cladding layer fluctuates the most.
(2) When the laser power is 1400 W, the cladding layer has the highest self-corrosion potential (−0.110 V), the lowest corrosion current (0.125 μA·cm^−2^), the largest polarization resistance (2,056,570 Ω), and the macroscopic performance is the best corrosion resistance. As the laser power increases, the Rct increases first and then decreases. When the laser power increases from 1000 W to 1800 W, the resistance value of Rct gradually increases. When the laser power is 1800 W, the cladding layer has a maximum charge transfer resistance Rct ((5.8864 ± 0.22) × 10^5^), which is much higher than that of the other three groups of experiments.
(3) The results of numerical simulation show that the current density of the β phase is larger than that of the α phase, and the β phase is more susceptible to corrosion. When the α phase and the β phase form a galvanic cell in the electrolyte, the potential of the β phase is lower than that of the α phase, so the electrolyte is always finitely dissolved. Due to the high potential of the α phase, it belongs to the protected party, and no corrosion occurs.

## Figures and Tables

**Table 1:** Physical properties of Ti-6Al-4V.

Density	Melting Point	Tensile Strength	Yield Strength	Coefficient of Linear Expansion
4.5 g/cm^3^	1660 °C	950 MPa	880 MPa	8.8 × 10^−6^/K

**Table 2:** Composition of TC4 alloy substrate.

Element	Al	V	Fe	O	Ti
Wt. %	5.5–6.76	3.5–4.5	≤0.25	≤0.2	Bal.

**Table 3:** Composition of TC4 alloy powder.

Element	Ti	Al	V	Fe	C	N	H	O
Wt. %	Bal.	5.5–6.8	3.5–4.5	≤0.3	≤0.1	≤0.05	≤0.015	≤0.20

**Table 4:** Analysis results of the dynamic polarization curves of the cladding layer and the matrix.

P/W	E_corr_/V	*i_corr_*/μA·cm^−2^	*β_a_*	*β_c_*	*R_p_*/Ω·cm^2^	*v*_corr_/mm·a^−1^
Sub	−0.469	0.247	5.887	4.101	237,669	3.019 × 10^−3^
1000	−0.221	0.664	8.879	5.665	2.165 × 10^6^	6.691 × 10^−4^
1400	−0.110	0.125	8.125	7.368	2.057 × 10^6^	9.299 × 10^−4^
1800	−0.118	2.970	5.893	6.564	1.784 × 10^6^	2.823 × 10^−4^
2200	−0.156	2.605	6.786	7.457	2.563 × 10^6^	2.356 × 10^−4^

**Table 5:** Equivalent circuit parameters obtained by fitting the EIS experimental results.

P/W	R_s_/ Ω·cm^−2^	(Q_1_ − Y)/ 10^−5^·Ω^−1^·s^−*n*^·cm^−2^	*n*_1_	R_d_/ 10^5^ Ω·cm^−2^	(Q_2_ − Y)/ 10^−5^·Ω^−1^·s^−*n*^·cm^−2^	*n*_2_	R_ct_/ 10^5^·Ω·cm^−2^
1000	66.16 ± 2.5	1.06 ± 0.08	0.86 ± 0.02	0.936 ± 0.05	(5.71 ± 0.12)	0.87 ± 0.01	4.5854 ± 0.15
1400	63.33 ± 2.1	3.75 ± 0.15	0.88 ± 0.01	1.323 ± 0.07	(5.65 ± 0.10)	0.84 ± 0.02	5.5496 ± 0.18
1800	64.98 ± 2.3	6.39 ± 0.20	0.89 ± 0.01	4.117 ± 0.12	(3.45 ± 0.08)	0.89 ± 0.01	5.8864 ± 0.22
2200	62.35 ± 2.0	1.00 ± 0.05	0.79 ± 0.02	3.797 ± 0.10	(4.48 ± 0.11)	0.77 ± 0.02	3.8318 ± 0.14

## Data Availability

The original contributions presented in this study are included in the article. Further inquiries can be directed to the corresponding author.

## References

[1] Zhao H., Zhao C., Xie W., Wu D., Du B., Zhang X., Wen M., Ma R., Li R., Jiao J. (2023). Research Progress of Laser Cladding on the Surface of Titanium and Its Alloys. Materials.

[2] Haochen L.I., Lin F.A., Haibing Z.H., Yingying W.A., Junlei T.A., Xuehan B.A., Mingxian S.U. (2022). Research Progress of Stress Corrosion Cracking of Ti-alloy in Deep Sea Environments. J. Chin. Soc. Corros. Prot..

[3] Hai M., Huang F., Wang Y. (2021). Brief Analysis of the application of titanium and titanium alloys in marine equipment. Metal World.

[4] Kothari K., Radhakrishnan R., Wereley N.M. (2012). Advances in gamma titanium aluminides and their manufacturing techniques. Prog. Aerosp. Sci..

[5] Weng F., Chen C., Yu H. (2014). Research status of laser cladding on titanium and its alloys: A review. Mater. Des..

[6] Cottam R., Brandt M. (2011). Laser cladding of Ti-6Al-4V powder on Ti-6Al-4V substrate: Effect of laser cladding parameters on mi-crostructure. Phys. Procedia.

[7] Afrasiabi M., Keller D., Lüthi C., Bambach M., Wegener K. (2022). Effect of process parameters on melt pool geometry in laser powder bed fusion of metals: A numerical investigation. Procedia CIRP.

[8] Jinlong W., Wenjie P., Tianlong L., Qingyuan W., Zeyu S., Gang Y. (2025). Fatigue failure analysis for marine propeller used titanium alloy under salty-water environment with the effect of corrosion durations. Eng. Fail. Anal..

[9] Nabhani M., Razavi R.S., Barekat M. (2019). Corrosion study of laser cladded Ti-6Al-4V alloy in different corrosive environments. Eng. Fail. Anal..

[10] Henry P., Takadoum J., Berçot P. (2009). Tribocorrosion of 316L stainless steel and TA6V4 alloy in H_2_SO_4_ media. Corros. Sci..

[11] Bayat M., Thanki A., Mohanty S., Witvrouw A., Yang S., Thorborg J., Tiedje N.S., Hattel J.H. (2019). Keyhole-induced porosities in Laser-based Powder Bed Fusion (L-PBF) of Ti6Al4V: High-fidelity modelling and experimental validation. Addit. Manuf..

[12] Paydas H., Mertens A., Carrus R., Lecomte-Beckers J., Tchuindjang J.T. (2015). Laser cladding as repair technology for Ti–6Al–4V alloy: Influence of building strategy on microstructure and hardness. Mater. Des..

[13] Liu X.B., Meng X.J., Liu H.Q., Shi G.L., Wu S.H., Sun C.F., Wang M.D., Qi L.H. (2014). Development and characterization of laser clad high temperature self-lubricating wear resistant composite coatings on Ti–6Al–4V alloy. Mater. Des..

[14] Ohidul Alam M., Haseeb A. (2002). Response of Ti-6Al-4V and Ti-24Al-11Nb alloys to dry sliding wear against hardened steel. Tri-Bol. Int..

[15] Molinari A., Straffelini G., Tesi B., Bacci T. (1997). Dry sliding wear mechanisms of the Ti6Al4V alloy. Wear.

[16] Li X.X., Li Y., Wang S. (2015). Study on the wear behavior and wear mechanism of TC4 alloy in different environmental media. Rare Met..

[17] Ren Z.Y., Hu Y.L., Tong Y., Cai Z.H., Liu J., Wang H.D., Liao J.Z., Xu S., Li L.K. (2023). Wear-resistant NbMoTaWTi high entropy alloy coating prepared by laser cladding on TC4 titanium alloy. Tribol. Int..

[18] Stern M., Geary A.L. (1957). Electrochemical polarization: I. A theoretical analysis of the shape of polarization curves. J. Electrochem. Soc..

[19] Chen J., Zhang Q. (2016). Effect of electrochemical state on corrosion–wear behaviors of TC4 alloy in artificial seawater. Trans. Nonferrous Met. Soc. China.

[20] Sawyer D.T., Day R.J. (1963). Kinetics for oxygen reduction at platinum, palladium and silver electrodes. Electrochim. Acta.

[21] Sato N., Sharma S.K. (2011). Green Corrosion Chemistry and Engineering.

[22] Hu P., Song R., Li X.J., Deng J., Chen Z.Y., Li Q.W., Wang K.S., Cao W.C., Liu D.X., Yu H.L. (2017). Influence of concentrations of chloride ions on electrochemical corrosion behavior of titanium-zirconium-molybdenum alloy. J. Alloys Compd..

[23] Linqing W., Yongtao Z., Junjun W., Zhongwei W.A., Weijiu H.U. (2019). Corrosion-wear interaction behavior of TC4 titanium alloy in simulated seawater. Tribology.

[24] Yang J., Song Y., Dong K., Han E.H. (2023). Research progress on the corrosion behavior of titanium alloys. Corros. Rev..

[25] Liu B.F., Hu J.N., Shi J.J., Gao X.W., Li J.Z. (2023). Effect of heat treatment on microstructure and corrosion resistance of TC4 titanium alloy manufactured by additive. Trans. Mater. Heat Treat..

[26] Ren J.Q., Li L., Wang Q., Xin C., Gao Q., Li J.C., Xue H.T., Lu X.F., Tang F.L. (2024). Effect of environmental media on the growth rate of fatigue crack in TC4 titanium alloy: Seawater and air. Corros. Sci..

[27] Zhao P., Song Y., Dong K., Shan D., Han E.H. (2021). Corrosion behavior of dual-phase Ti–6Al–4V alloys: A discussion on the impact of Fe content. J. Alloys Compd..

[28] Dong Y., Huang S., Wang Y., Zhang B., Alexandrov I.V., Chang H., Dan Z., Ma L., Zhou L. (2022). Stress corrosion cracking of TC4 ELI alloy with different microstructure in 3.5% NaCl solution. Mater. Charact..

